# Clinical use of linezolid in periprosthetic joint infections – a systematic review

**DOI:** 10.5194/jbji-6-7-2020

**Published:** 2020-07-13

**Authors:** Christoph Theil, Tom Schmidt-Braekling, Georg Gosheger, Jan Schwarze, Ralf Dieckmann, Kristian Nikolaus Schneider, Burkhard Möllenbeck

**Affiliations:** Department of Orthopedics and Tumor Orthopedics, Muenster University Hospital, Albert-Schweitzer-Campus 1, 48149, Münster, Germany

## Abstract

**Introduction**: The most common causative organism in periprosthetic joint infections (PJIs) is Gram-positive bacteria that are increasingly drug resistant. In these cases the use of linezolid may be warranted. However, there are conflicting reports regarding its role in antibiotic treatment of PJIs. The aim of this review is to gather and analyze clinical results and treatment details on linezolid in patients with PJIs. **Methods**: In August 2019, a comprehensive literature search using MEDLINE (Pubmed and Ovid) and Cochrane Library was performed. A total of 504 records were screened, and a total of 16 studies including 372 patients treated with linezolid for a PJI were included in this review based on the PRISMA criteria and after quality analysis using the MINOR score and Newcastle–Ottawa scale, as well as assessing level of evidence. Pooling analysis as well as descriptive analysis was performed. **Results**: Based on the results from the studies included, infection control was achieved in 80 % (range 30 %–100 %) of patients after a mean follow-up period of 25 (range 2–66) months. The mean duration of treatment was 58 d intravenous and orally at a median dose of 600 mg bis in die (b.i.d.) (range 400–900 b.i.d.). A combination therapy with rifampicin was used in 53 % of patients. MRSA (methicillin-resistant *Staphylococcus aureus)* infections were present in 29 % and resistant CoNS (coagulase-negative *Staphylococcus*) in 46 %. Adverse effects occurred in 33 % of cases, mostly anemia, thrombocytopenia and gastrointestinal complaints leading to treatment discontinuation in 9 %. However, great heterogeneity was found with respect to surgical treatment, diagnosis of infection and indication for linezolid. **Discussion**: Linezolid is an appropriate option for treatment of resistant Gram-positive organisms in PJIs. Most commonly 600 mg b.i.d. is used, and a combination with rifampicin appears feasible although one must consider individual increases in doses in these cases. However, adverse effects are common and there are limited data for long-term use and optimal antibiotic combinations or individual doses.

## 1 Introduction

The treatment of periprosthetic joint infection (PJI) includes different surgical approaches involving debridement and prosthesis retention as well as one-stage exchange or two-stage exchange, and medical systemic treatment can vary greatly regarding length and substances used (Osmon et al., 2013b; Anemuller et al., 2019; de Beaubien et al., 2019) with successful shorter-term courses (Winkler et al., 2019) described as being contrasted by long-time antibiotic suppression treatment in severe, complicated cases (Siqueira et al., 2015; Wouthuyzen-Bakker et al., 2017; Leijtens et al., 2019). Furthermore, microbiological results are changing with increasing prevalence of resistant strains (Drago et al., 2017; De Vecchi et al., 2018), particularly methicillin-resistant (MR) coagulase-negative *Staphylococcus* (CoNS) now being the main pathogen detected (Lourtet-Hascoet et al., 2018; Hipfl et al., 2019; Tevell et al., 2019). In this context, linezolid is a potential antimicrobial treatment option addressing resistant *Staphylococcus* (Deroche et al., 2019) as well as reducing the need for long-term inpatient treatment given its excellent oral bioavailability (Kutscha-Lissberg et al., 2003). However, linezolid can have some feared adverse effects such as cytopenia, particularly of leukocytes and neuropathy, that might lead to treatment discontinuation (Legout et al., 2010). Furthermore, due to biofilm formation on the infected implant by *Staphylococcus*, rifampicin as a biofilm-active drug (Zimmerli and Sendi, 2019) could potentially be combined with linezolid in these infections. However, there are concerns regarding adverse effects and drug interactions with this combination (Gomez et al., 2011; Gandelman et al., 2011).

While current consensus statements and widely used treatment guidelines recommend the use of linezolid only for (vancomycin-)resistant *Enterococcus* or as an alternative treatment for resistant *Staphylococcus* (Anemuller et al., 2019; de Beaubien et al., 2019; Osmon et al., 2013b), there are several reports that recommend its use as either an empirical treatment (Deroche et al., 2019; Takoudju et al., 2018) or for early oral treatment reducing the need for in-hospital intravenous treatment with good results (Oussedik and Haddad, 2008; Legout et al., 2010; Cobo et al., 2013). Furthermore, in implant-related infections rifampicin and its derivatives needs to be considered as a potential drug for combined treatment considering its anti-biofilm properties in *staphylococcal *infections (Zimmerli and Sendi, 2019).

A previous review article on linezolid in orthopedic implant infections (Morata et al., 2014a) reported a success rate of around 70 % with adverse effects reported to occur in 34 % of all cases. However, orthopedic implant infections included in this analysis range from very minor infections such as external fixator pin infections to severe prosthetic (re-)infections for which surgical treatment, patient characteristics and common length of treatment as well as success rates are expected to vary greatly (Aboltins et al., 2019; Metsemakers et al., 2018; Moriarty et al., 2016). To our knowledge, there is no review on the use of linezolid for prosthetic joint infections specifically.

The aim of this review is to evaluate the study quality of published articles and to analyze current clinical results as well as treatment details and microbiology findings of PJIs treated with linezolid.

## 2 Methods

**Figure 1:**
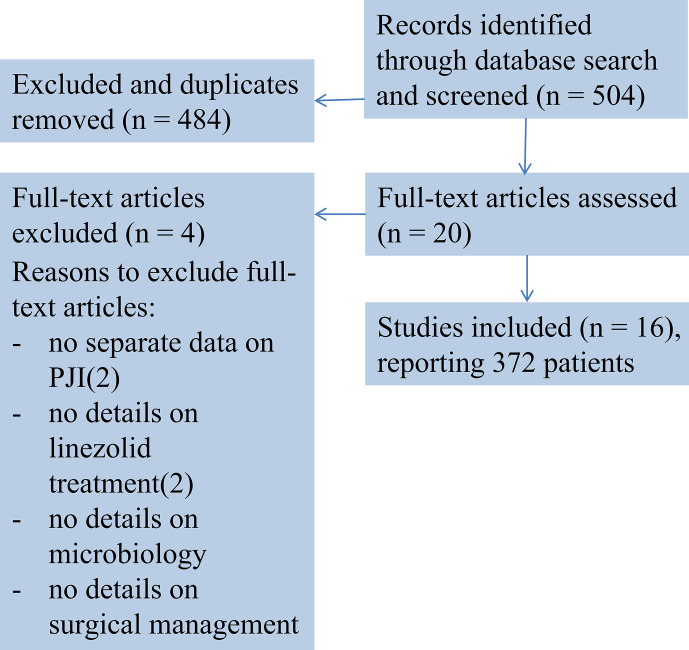
Flowchart showing the studies included.

A comprehensive literature research of publications until 12 August 2019 using the Pubmed, Ovid Embase and Cochrane Library search was performed. Search terms were “linezolid periprosthetic/prosthetic joint/s infection/s”, “linezolid joint/s infection/s”, “linezolid joint/s”, “linezolid bone” and “linezolid arthroplasty/ies”. The search was restricted to studies on humans published between 1950 and August 2019 for papers in English. The review algorithm was based on the Preferred Reporting Items for Systematic Review and Meta-Analyses (PRISMA) criteria (Moher et al., 2009), and search results are presented in a PRISMA conform diagram (Fig. 1).

Titles and abstract were reviewed by two authors (Christoph Theil and Burkhard Möllenbeck). Following exclusion based on title and abstract, a full text was obtained and reviewed by the same investigators. In many cases supplemental materials were obtained and reviewed additionally. The study quality was assessed using the Methodological Index for Non-Randomized Studies (MINORS) checklist (Slim et al., 2003) that allows for the calculation of a quality score (maximum score out of 16 for observational studies and out of 24 for comparative studies) as well as the Newcastle–Ottawa scale (ranging from 0 to 9 stars). The level of evidence was determined using the Oxford Centre for Evidence-Based Medicine (OCEBM) levels of evidence (OCEBM Levels of Evidence Working Group, 2011).

Inclusion criteria were the use of linezolid in PJIs of hip and knee joint replacements. Case reports, reviews on PJIs and studies with fewer than five patients were excluded. Studies about orthopedic infections that mention linezolid use in PJIs but in which results for treatment of PJIs could not be extracted were excluded (four studies). If data on PJIs were presented among other data on infections in which all patients were treated with linezolid, it was extracted and general statements (e.g., median age or follow-up period) were assumed to be applicable for patients treated with PJIs in these studies. These results are highlighted. Further references were obtained by reviewing general practice guidelines and consensus statements (Osmon et al., 2013a, b; de Beaubien et al., 2019) regarding the use of linezolid in PJIs.

Microbiology details, length of treatment, potential antibiotic combinations as well as adverse effects have been extracted from the studies. The primary outcome measure was infection control as defined by the respective study. Despite the heterogeneity of studies and missing values for several variables obtained from the studies presented in terms of surgical management, definition of infection as well as administration and length of antibiotic treatment, we chose to perform a simple pooling of the aggregate results for the outcome measures when possible and a descriptive analysis. Descriptive statistics were used to analyze distribution of data. Weighed (based on the number of cases per study included in the analysis of different outcome measures) means and ranges were calculated for parametric data and medians and interquartile ranges (IQRs) for nonparametric data. Due to the heterogeneity encountered no further statistical analysis or meta-analysis was performed.

Statistical analysis was performed using Microsoft Excel 2016 (Microsoft Corporation, Redmond, Washington, USA) and SPSS Statistics for Windows Version 25 (IBM Corporation, Armonk, New York, USA).

## 3 Results

### 3.1 Study types and quality

We were able to identify a total of 16 studies that met the inclusion criteria. The studies included in this article report the outcome of a total of 372 patients treated with linezolid for a PJI.

There were no randomized controlled studies, and all studies were of observational nature. Nine studies were retrospective single or multicenter studies while seven studies were prospective single or multicenter studies reporting results on a mean of 16 patients (range 8–53) with a mean age of 64 years (range 54–76) after a mean follow-up period of 25 months (range 2–66) (Table 1).

**Table 1:** Study quality and methodological assessment.

	Design	No. of patientsincluded	Follow-up period in months	MINORS score (out of 16 if not otherwise indicated)	Newcastle–Ottawa score	Level of evidence (Oxford)
Cobo et al. (2013)	prospective, multicenter	25	14	13	5	3
Bassetti et al. (2005)	retrospective, single center	20	12	11	5	4
Gomez et al. (2011)	prospective, single center	49	24	14	6	3
Harwood et al. (2006)	prospective, single center	11	13	8	5	4
Joel et al. (2014)	retrospective, single center	10	34	12	5	4
Legout et al. (2010)	retrospective, multicenter	39	16	10	7	3
Lu et al. (2010)	prospective, multicenter	17	6	8	5	4
Morata et al. (2014a)	retrospective, multicenter	38	25	18/24	7	3
Nguyen et al. (2009)	retrospective, multicenter	11	24	17/24	7	3
Oussedik and Haddad (2008)	retrospective, single center	14	33	12	6	4
Papadopoulos et al. (2009)	prospective, case-control study	8	2	12	7	3
Rao and Hamilton (2007)	prospective, single center	23	19	11	5	3
Razonable et al. (2004)	retrospective, single center	8	7	10	5	4
Soriano et al. (2007)	prospective, multicenter	53	Min. 12–47${}^{*}$	14	7	3
Tornero et al. (2016)	retrospective, single center	17	66${}^{*}$	18/24	7	3
Eriksson et al. (2019)	retrospective, single center	28	51.6	12	6	4

${}^{*}$ No PJI-specific results.

### 3.2 Infection control

The infection was initially controlled in 80 % (229/285) of cases included for that measure with three studies only reporting a range (Harwood et al., 2006; Rao and Hamilton, 2007; Soriano et al., 2007) (Table 1) because of heterogeneous patient cohorts. Additionally, five studies reported a separate median reinfection rate during the respective follow-up period (Bassetti et al., 2005; Cobo et al., 2013; Eriksson et al., 2019; Morata et al., 2014a; Oussedik and Haddad, 2008) with a mean of 22 % (26/120 patients). Based on the available information and different definitions, 48 % (range 0–100 %) of all PJIs can be considered early infections (119/244) and 52 % of all PJIs were infected TKA (total knee arthroplasty) (112/217).

### 3.3 Indications, treatment details and microbiological details

The indications for the use of linezolid varied between the studies. Two studies included all patients with Gram-positive PJIs (Bassetti et al., 2005; Cobo et al., 2013). Six studies reported resistant bacteria as an indication (Eriksson et al., 2019; Morata et al., 2014a; Nguyen et al., 2009; Papadopoulos et al., 2009; Rao and Hamilton, 2007; Razonable et al., 2004). Failure or intolerance of previous treatment was noted in nine studies (Eriksson et al., 2019; Harwood et al., 2006; Gomez et al., 2011; Legout et al., 2010; Lu et al., 2010; Rao and Hamilton, 2007; Razonable et al., 2004; Soriano et al., 2007; Papadopoulos et al., 2009) or oral application following intravenous treatment in two studies (Joel et al., 2014; Oussedik and Haddad, 2008). The mean length of treatment in the included studies (combined intravenously and orally) was 58 d (25–125 d). Prior to the use of linezolid, five studies reported a different intravenous treatment (Eriksson et al., 2019; Gomez et al., 2011; Harwood et al., 2006; Joel et al., 2014; Oussedik and Haddad, 2008) or antibiotic combined treatment for polymicrobial infections in up to 35 % of patients (Legout et al., 2010). With regard to the role of rifampicin–linezolid combinations, eight studies report no parallel use of rifampicin and linezolid, while on the other hand eight studies (Legout et al., 2010; Joel et al., 2014; Eriksson et al., 2019; Soriano et al., 2007; Tornero et al., 2016; Morata et al., 2014a; Gomez et al., 2011; Nguyen et al., 2009) generally use this combination when sensitive organisms and *Staphylococcal* infection were present in a mean of 53 % of cases (131/246 patients, range 3 %–100 %) in the respective studies.

As expected the most commonly isolated microorganism was *Staphylococcus*. Six studies (Harwood et al., 2006; Lu et al., 2010; Bassetti et al., 2005; Razonable et al., 2004; Legout et al., 2010; Nguyen et al., 2009) reported methicillin-resistant *Staphylococcus aureus* (MRSA) as the main pathogen in a mean of 29 % (range 0 %–85 %) of cases, while otherwise coagulase-negative* Staphylococcus* was the most common organism with a mean of 46 % (range 15–100) of patients. Five studies also used linezolid in culture-negative infections (Gomez et al., 2011; Papadopoulos et al., 2009; Harwood et al., 2006; Soriano et al., 2007; Lu et al., 2010), mostly as a second-line treatment, with a median percentage of culture-negative infections in these studies of 14 % (IQR 11 %–25 %). On the other hand polymicrobial infection was reported in 11 studies (Joel et al., 2014; Tornero et al., 2016; Morata et al., 2014a; Razonable et al., 2004; Legout et al., 2010; Soriano et al., 2007; Cobo et al., 2013; Oussedik and Haddad, 2008; Harwood et al., 2006; Rao and Hamilton, 2007; Nguyen et al., 2009) with a median percentage of 8 % of patients included (IQR 2 %–37 %).

Microbiology findings, treatment details and antibiotic duration are summarized in Table 2.

**Table 2:** Systemic and local treatment details, microbiological findings.

Study	Type of infection	Indication linezolid	Duration andtreatmentdetails	Linezolid dose	Combination with other antibiotics	Surgicalmanagement	Microbiology
Cobo et al. (2013)	chronic	all Gram-positiveinfections	42 d (IV or oral)	600 b.i.d.	permitted antibiotic combinations for polymicrobial infection, long-term clindamycin in one case	two-stage	81 % *Staphylococcus*, 19 % *Streptococcus* and others, no MRSA, 33 % MRSE PM 8 % CN: 0 %
Bassetti etal. (2005)	45 % acute55 % chronic	all Gram-positive infections	50.4 d (IV and oral)	not reported	75 % patients previous treatment, 55 % ciprofloxacin–rifampicin combination, 20 % glycopeptide	DAIR or single stage	70 % MRSA, 25 % MRSE, 5 % *Enterococcus* PM 0 % CN: 0 %
Gomez etal. (2011)	63 % early (<30 d) 37 % late (>30 d)	failed prior treatment	80.2 d oral	600 b.i.d.	100 % combination with rifampicin, otherwise ciprofloxacin, teicoplanin, cotrimoxazole	77.8 % DAIR, 22.8 % non-operative	45 % MRSE, 12 % MRSA PM 0 % CN: 35 %
Harwood et al. (2006)	n/a	intolerance of gly-copeptide, failedprior treatment, oral continuation	39 d oral${}^{*}$	not reported	previous or combination: flucloxacillin, cephalosporin,vancomycin, rifampicin	18 % non-operative, 27 %DAIR, 54 %two-stage	85 % MRSA, 15 % CoNS${}^{*}$ PM 6 % CN: 2 %
Joel et al.(2014)	n/a	oral continuation oftherapy	30 d oral	not reported	previous treatment: flucloxacillin, cephalosporin,vancomycin, rifampicin	n/a	70 % CoNS, 10 % MRSA, 10 % *Enterococcus*, 10 % *Staphylococcusaureus* PM 0 % CN: 11.3 %
Legout et al. (2010)	n/a	contraindications for other, vancomycinintolerance	101.5 d oral${}^{*}$	600 b.i.d.	combination with fluoroquinolones, beta-lactams, others	DAIR, onestage, two-stage	36 % MRSA, 21 % MRSE, 6 % Enterococcus${}^{*}$ PM 38 % CN: 0 %
Lu et al.(2010)	n/a	failure or intolerance of other treatment	25 d IV and oral${}^{*}$	600 b.i.d.	30 with vancomycin and 25 with teicoplanin, and 5 cases received fusidic acid, 2 gentamicin, 2 ciprofloxacin, 1 trimethoprim/sulfamethoxazole, 1 cefazolin, 1 rifampicin, and 1 oxacillin	n/a	79 % MRSA, 9.4 % MSSA${}^{*}$ PM 0 % CN: 11.3 %
Morata et al. (2014a)	90 % acute(<4 weeks), 10 % late acute	n/a	44.5 d IV and oral	600 b.i.d., up to 900 b.i.d. withrifampicin	ciprofloxacin, beta-lactam for polymicrobial infection	DAIR	61 % CoNS, 13 % MRSA, 7 % *Enterococcus* PM 38 % CN: 0 %
Nguyen etal. (2009)	chronic >30 d of infection, >2 months postoperatively	Gram-positive coccalinfection	124.6 d IV and oral${}^{*}$	600 b.i.d.	glycopeptide, cephalosporin	27 % one-stage, 27 % two-stage, 36 % DAIR,9 % resection arthroplasty	34.4 % MRSA, 28.1 % CoNS, 15.6. % Enterococcus${}^{*}$ PM 3.1 % % CN: 0 %
Oussedik and Haddad (2008)	71 % chronic, 29 %early or intermediate	oral treatment	37.1 d oral	600 b.i.d.	Teicoplanin	85 % two-stage, 7 % DAIR, 7 % one-stage	57 % CoNS, 29 % MRSA, 15 % MSSA PM 7 % CN: 0 %
Papadopoulos et al. (2009)	n/a	resistant bacteria, intolerance of glycopeptide	42 d i.v and oral	600 b.i.d.	none	62.5 % non-operative, 37.5 % stagedrevisions	50 % MRSE, 25 % MRSA PM 0 % CN: 25 %
Rao and Hamilton (2007)	n/a	intolerance, failure, resistance to vancomycin	42 d IV and oral	600 b.i.d.	possible for Gram-negative or fungal infection, suppression therapy in selected cases using cephalexin, minocycline, trimethoprim, fluoroquinolones	61 % DAIR,39 % staged revision	39 % MRCoNS, 22 % MRSA, 2 *Enterococcus* PM 4 % CN: 0 %
Razonable et al. (2004)	63 % chronic, 37 % acute	vancomycin resis-tance, intolerance ofvancomycin, failure ofvancomycin	49 d oral	600 b.i.d., lowered to400 b.i.d. intwo patients	fluoroquinolones, cephalosporin, beta-lactam, fluconazole depending onmicrobiology	75 % resection arthroplasty, 25 % DAIR	50 % MRSA, 50 % CoNS, 25 % VRE PM 37 % CN: 0 %
Soriano et al. (2007)	28 % acute, 72 % chronic	oral continuation oftherapy, failure orintolerance of previous treatment	56 d oral${}^{*}$	600 b.i.d.	in polymicrobial infections	63 % implant retention${}^{*}$, 37 % stagedrevision	n/a PM 16 % CN: 14 %
Tornero etal. (2016)	acute (within 90 d)	n/a	76 d IV and oral${}^{*}$	600 b.i.d.	vancomycin and ceftazidime, rifampicin in 8 out of 15 cases	DAIR	CoNS 48 %, *Staphylococcus aureus*37 %, *Enterococcus* 13 %${}^{*}$ PM 39 % CN: n/a
Eriksson et al. (2019)	58 % early (<3 months), 39 % delayed (3–24 months), 3 % late (>24 months)	intolerance of other treatment (5/28), resistant CoNS	29.4 d oral	600 b.i.d.	vancomycin 22/28, teicoplanin 3/28, cloxacillin 2/28, daptomycin 1/28	54 % 2-stage exchange, 46 % DAIR	CoNS (16/28) PM n/a CN: 0 %

PM – polymicrobial; CN – culture negative; CoNS – coagulase-negative *Staphylococcus*; DAIR – debridement, antibiotics, irrigation, retention; n/a – not available; MRSE – methicillin-resistant CoNS; MSSA – methicillin-sensitive *Staphylococcus aureus*; VRE – vancomycin-resistant *Enterococcus*;MRCoNS – methicillin-resistant coagulase-negative *Staphylococcus*.${}^{*}$ No PJI-specific results.

Adverse effects were reported in 94 % (15/16) of studies included. The mean frequency of adverse events was 33 % (range 7 %–76 %). The most common complications were hematological alterations; 75 % (12/16) of studies included report a percentage of patients who discontinued treatment at a mean rate of 9 % (range 0 %–44 %) (Table 3).

**Table 3:** Adverse effects reported.

Study	% ofadverse effects	% ofdiscontinuation	Types of adverse effects
Cobo et al. (2013)	76 %	13 %	76 % thrombocytopenia 40 % nausea 36 % anemia
Bassetti et al. (2005)	15 %	0 %	15 % gastrointestinal symptoms none hematological
Gomez et al. (2011)	36.6 %	0 %	12 % candidiasis and gastrointestinal discomfort 6 % thrombocytopenia 6 % anemia
Harwood et al. (2006)	44 %	19 %${}^{2}$	15 % anemia 15 % nausea vomiting 11 % diarrhea
Joel et al. (2014)	10 %	10 %	10 % thrombocytopenia
Legout et al. (2010)	48 %	15 %${}^{*}$	48 % thrombocytopenia 29 % anemia 9 % neuropathy
Lu et al. (2010)	25 %	11.3 % discontinued${}^{*}$	25 % thrombocytopenia 18 % anemia 6 % leukopenia no neuropathy
Morata et al. (2014a)	38 %	0 %	26 % gastrointestinal 13 % hematological 5 % neurotoxicity
Nguyen et al. (2009)	42.9 %	14.3 %${}^{*}$	14 % anemia 14 % gastrointestinal 7 % hepatic enzyme elevation
Oussedik and Haddad (2008)	7 %	0 %	7 % pancytopenia
Papadopoulos et al. (2009)	33 %	44 %${}^{*}$	33 % anemia 9 % thrombocytopenia 6 % GIT symptoms no neuropathy
Rao and Hamilton (2007)	22 %	13 %	17 % thrombocytopenia 9 % GIT symptoms 9 % anemia
Razonable et al. (2004)	50 %	0 %	50 % leukopenia 25 % thrombocytopenia 13 % neuropathy
Soriano et al. (2007)	13 %	0 %	12.9 % GI symptoms 4.7 % thrombocytopenia 5.8 % anemia no neuropathy${}^{*}$
Tornero et al. (2016)	n/a	n/a	n/a
Eriksson et al. (2019)	39 %	14 %	21 % anemia 7 % thrombocytopenia 4 % leukopenia

PM – polymicrobial; CN – culture negative; CoNS – coagulase-negative *Staphylococcus*; DAIR – debridement, antibiotics, irrigation, retention; GIT – gastrointestinal; n/a – not available. ${}^{*}$ No PJI-specific results.

## 4 Discussion

Linezolid offers the advantage of very good, oral bioavailability (Thompson et al., 2017) and its broad spectrum against Gram-positive bacteria facing current resistance patterns (Deroche et al., 2019; Lourtet-Hascoet et al., 2018) of organisms encountered in the treatment of PJI. Based on the reports available and included in this review a remission of the infection can be expected in around 80 % of cases. However, there are several issues that surgeons and infectious disease specialists need to consider in this environment.

While this review reports on a large number of patients treated with linezolid and the results presented can help in planning antimicrobial treatment as it provides an overview about adverse effects, expected rates of infection control and potential antibiotic combinations, there are limitations to this study. As with most reviews, we relied on published data and had several missing values for some variables due to this. We still chose to pool some of the data provided despite heterogeneity of the different studies regarding the indication, definition of infection and surgical treatment as this can be assumed to be the case in everyday practice. As the most common treatment approach for which data could be extracted was implant retention for early or acute infection, we chose to present the results of these patients separately and pool the results regarding outcome. However, even for “early” infections, the definition varies across studies from patients having symptoms of infection for only 2–3 weeks in some studies and up to 3 months following primary surgery in others (Tornero et al., 2016). A further limitation that needs to be considered when interpreting the results of the studies included in this review is that one main indication for the use of linezolid reported was failure of the previous treatment. Given that repeat staged prosthetic revisions and further septic surgeries following treatment of a PJI are known to lead to much worse results (Kheir et al., 2017; Khan et al., 2019) with regard to remission of an infection, the results discussed here might be low-end estimates of the potential success rate when using linezolid in PJI patients. However, this might reflect current recommendations regarding the use of linezolid as a second-line reserve therapy (Osmon et al., 2013b; Aboltins et al., 2019; Sendi and Zimmerli, 2012). Furthermore, antibiotic susceptibilities might change (Tevell et al., 2019) even in the short-term between treatment stages of a two-stage exchange (George et al., 2018) potentially necessitating the use of linezolid due increased resistance in staged interventions if further revision is required.

In a previous review article Morata et al. (2014b) differentiated treatment success using linezolid based on the surgical approach, which is certainly an important factor that needs to be taken into account when comparing the results of a specific drug presented by different authors. Soriano et al. (2007) for instance reported remission in 38.7 % of chronic PJIs treated with implant retention versus 83 %–100 % remission using a staged implant exchange while treating both groups with linezolid. Future studies should focus on reporting results using current uniform definitions for infection (Parvizi et al., 2018; Signore et al., 2019) as well as standardized treatment algorithms including details on surgical and medical treatment (Sendi and Zimmerli, 2012).

The most common organism isolated when linezolid was used is resistant specimens of *Staphylococcus aureus *and coagulase-negative *Staphylococcus*. Considering the general consensus that rifampicin and its derivatives play a vital role in combating the biofilm on implants (Zimmerli and Sendi, 2019) when treating PJIs, linezolid needs to be evaluated with regard to linezolid–rifampicin-based combination treatment. While an in vitro study (Thompson et al., 2017) suggested the use of such an oral-only treatment regiment, there is conflicting evidence regarding clinical data on rifampicin containing regimens. While Legout et al. (2010) found a lower incidence of anemia when combining rifampicin and linezolid with no difference in remissions, there are two clinical studies (Morata et al., 2014a; Tornero et al., 2016) from one institution that found that a combination treatment was associated with a higher rate of relapse and ultimately treatment failure compared to a linezolid monotherapy or other quinolone-based combinations. This effect is potentially due to the interaction in the cytochrome P-based metabolism of linezolid that is increased when rifampicin is added (Gandelman et al., 2011). The available concentration of linezolid might therefore drop beneath the respective minimal inhibitory concentration (MIC) needed to eliminate the bacteria (Tornero et al., 2016). Therefore, while a combination of linezolid and rifampicin might be desirable in staphylococcal PJI, it has potential adverse effects on the desired control of infection. For future studies, different dosing regimens of linezolid could be evaluated given that some patients appear to be at subtherapeutic levels with the standard dose (Pea et al., 2010), and there currently is no study on the pharmacokinetics of linezolid in patients treated for PJI. In this context, rifampicin could be reevaluated as a useful partner for linezolid.

The optimal antibiotic treatment length in PJI is currently unknown (Aboltins et al., 2019). However, long-term or life-long treatment algorithms were recommended by some guidelines and authors (Leijtens et al., 2019; Osmon et al., 2013b; Calabro et al., 2019; Aboltins et al., 2019), and duration of treatment might play a vital role (Tattevin et al., 2006) – raising the question of whether linezolid, given its adverse effects, is a potential option for long-term treatment or even suppression. In a study on chronic infections not limited to orthopedic infections, Vazquez et al. (2016) concluded that with monitoring of adverse effects, a long-term treatment of greater than 6 weeks can be safely performed. Additionally, therapeutic drug monitoring has proven effective in optimizing dosing regiments in non-orthopedic infection (Pea et al., 2012) and should be implemented in the treatment of PJIs as well, particularly considering that some patients might benefit from dose escalation or de-escalation to ensure adequate MICs and potentially reduce the high percentage of therapy discontinuation in PJI patients reported by some authors (Legout et al., 2010; Harwood et al., 2006).

Other options in the treatment of Gram-positive infections include aminoglycosides, which have the additional advantage of providing excellent elution characteristics from bone cement and can be used as a local treatment, particularly combined with glycopeptides (Badha et al., 2019). However, as a relevant amount of these drugs might be absorbed, systemic complications must be monitored for at least 8 weeks especially if systemic treatment is performed as well (Edelstein et al., 2018). Furthermore, patients with repeat revision or reinfection might be at high risk of developing resistant strains when aminoglycosides were used in a previous surgery (Corona et al., 2014).

In conclusion, despite its long-term use, potential in combating increasingly resistant *Staphylococcus *and generally successful results, there are still several questions that need to be answered regarding the role of linezolid, its optimal treatment modality and potential antibiotic combination. Longer courses of treatment require close surveillance, and patients at risk of non-optimal dosage should undergo drug monitoring.

## Data Availability

All underlying data are in the text and tables.
